# Translocation Renal Cell Carcinoma: An Update on Clinicopathological and Molecular Features

**DOI:** 10.3390/cancers9090111

**Published:** 2017-08-29

**Authors:** Kentaro Inamura

**Affiliations:** Division of Pathology, The Cancer Institute, Japanese Foundation for Cancer Research, 3-8-31 Ariake, Koto-ku, Tokyo 135-8550, Japan; kentaro.inamura@jfcr.or.jp; Tel.: +81-3-3570-0111 (ext. 5604); Fax: +81-3-3570-0558

**Keywords:** ALK, fusion, kidney, renal cell carcinoma, TFE3, TFEB, translocation

## Abstract

Microphthalmia-associated transcription (MiT) family translocation renal cell carcinoma (tRCC) comprises Xp11 tRCC and t(6;11) RCC. Due to the presence of fusion genes, Xp11 tRCC and t(6;11) RCC are also known as *TFE3*- and *TFEB*-rearranged RCC, respectively. TFE3 and TFEB belong to the MiT family, which regulates melanocyte and osteoclast differentiation, and *TFE3*- and *TFEB*-rearranged RCC show characteristic clinicopathological and immunohistochemical features. Recent studies identified the fusion partner-dependent clinicopathological and immunohistochemical features in *TFE3*-rearranged RCC. Furthermore, RCC with chromosome 6p amplification, including *TFEB*, was identified as a unique subtype of RCC, along with *ALK*-rearranged RCC. This review summarizes these recent advancements in our tRCC-related knowledge.

## 1. Introduction

Renal cell carcinoma (RCC) represents a group of molecularly heterogeneous diseases characterized by differing sets of genetic and epigenetic abnormalities [1,2,3,4,5,6,7,8,9,10,11,12,13,14,15,16,17,18,19]. Microphthalmia-associated transcription (MiT) family translocation renal cell carcinoma (tRCC) is an RCC subtype characterized by early onset. The MiT family of transcription factors—including MiTF, TFE3, TFEB, and TFEC—shares a basic helix-loop-helix (bHLH) DNA-binding domain and similar target genes. In addition to MiT family tRCC, alveolar soft part sarcoma, melanoma, clear cell sarcoma, angiomyolipoma, and perivascular epithelioid cell tumor (PEComa) highly express MiT family transcription factors as well as show common morphological, immunohistochemical, and molecular features, including *TFE3* rearrangement; *TFE3* gene fusions have been identified in PEComas of the kidney and soft tissue, including those demonstrating melanin pigments [20,21]. This review provides updated information gained from new cases of MiT family tRCC [1] and summarizes *ALK*-rearranged RCC, an emerging RCC subtype that may be treatable by ALK-targeted therapy.

## 2. New Category of MiT Family tRCC

Xp11 tRCC was originally described by Argani’s group [22,23,24] and established as an RCC subtype in the 2004 WHO classification. In the 2016 WHO classification [1], MiT family tRCC, comprising Xp11 tRCC and t(6;11) RCC, was newly defined as an RCC subtype. Xp11 and t(6;11) RCC are characterized by the rearrangement of the MiT transcription factors *TFE3* and *TFEB*, respectively. Although the majority of RCCs can be diagnosed with only a morphological assessment, MiT family tRCC also requires the confirmation of *TFE3* or *TFEB* rearrangement. The common fusion partners of *TFE3* are *ASPSCR1* (also known as *ASPL*), *PRCC*, *SFPQ* (also known as *PSF*), and its rare fusion partners include *CLTC*, *NONO*, *RBM10*, *PARP14*, *LUC7L3*, *KHSRP*, *DVL2*, *MED15*, and *GRIPAP1*. The fusion partner of *TFEB* in t(6;11) RCC is *MALAT1* (also known as *Alpha*). t(6;11) RCC has a fusion of *TFEB* in 6p21 with *MALAT1* in 11q12. *MALAT1* is a well-known long non-coding RNA (lncRNA) that fuses to *TFEB* upstream of the translation initiation codon ATG in exon 3. Therefore, the fusion transcript of *MALAT1-TFEB* encodes full length *TFEB*.

The comprehensive molecular characterization by the Cancer Genome Atlas (TCGA) research network identified two new fusion partners of *TFEB* (*COL21A1-TFEB* and *TFEB-CADM2*) in the analysis of papillary RCC [3]. Malouf et al. identified a novel fusion partner of *TFEB* (*TFEB-KHDRBS2*) in the TCGA database of clear cell RCC [2,25]. However, these three new fusion genes in the TCGA database are likely passenger genes. The RCCs with these three fusions had not only *TFEB* rearrangement but also amplification of 6p, which is where *TFEB* is located. Recently, RCC with 6p amplification was shown to be a unique RCC subtype with characteristic histological features and aggressive behavior [26,27,28].

## 3. Clinical Characteristics of MiT Family tRCC

Xp11 tRCC comprises 20–40% of childhood RCC and 1–4% of adult RCC with an average age of onset of 50 years [29,30]. t(6;11) RCC is very rare with approximately 60 cases reported thus far. The average age of onset of t(6;11) RCC is approximately 30 years old. However, adult t(6;11) RCCs have been identified by us (Case 1, 57-year-old Japanese man) [31] and others [32,33]. Importantly, adults both young and old can suffer from t(6;11) RCC. The original cases of Xp11 tRCC demonstrated indolent behaviors [22,23,24]; however, Xp11 tRCC frequently shows lymph node metastasis and has a worse prognosis than papillary RCC and similar prognosis with clear cell RCC [30]. Xp11 tRCCs have the potential to metastasize as late as 20–30 years after diagnosis. Among Xp11 tRCCs, Xp11 tRCC with an *ASPSCR1-TFE3* fusion, which is also detected in alveolar soft part sarcoma, was reported to have a worse prognosis than Xp11 tRCCs with other fusion partners [34]. Xp11 tRCC in childhood patients is generally considered to have a better prognosis [34]. t(6;11) RCC is also considered to have a good prognosis, but the number of reported cases is too small to reach a definitive answer. Indeed, lethal t(6;11) RCC with aggressive behavior was reported (Case 2, 37-year-old Japanese man) [31]. For childhood MiT tRCCs, chemotherapy is a known risk factor [35]. Because MiT family tRCCs express higher levels of phosphorylated S6, which correlates positively with the activation of the mTOR pathway, than most other RCC subtypes, mTOR inhibitors may be a specific therapeutic drug for MiT family tRCC [32,36].

## 4. Pathological Characteristics of MiT Family tRCC

### 4.1. Xp11 tRCC

Grossly, Xp11 tRCC presents as a brownish-yellow solid mass with frequent necrosis and hemorrhage, similar to clear cell RCC. Occasionally, Xp11 tRCC resembles papillary RCC with a gray-white cut surface.

**Figure 1:**
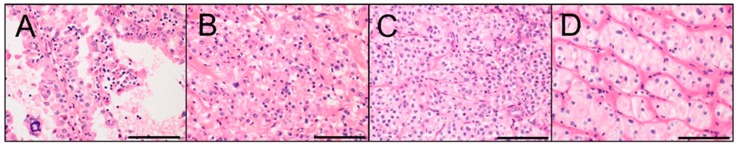
Morphology of MiT family tRCC (Hematoxylin and Eosin staining; scale bar, 100 µm). (**A**) Xp11 tRCC, (**B**) t(6;11) RCC (Case 1), (**C**) t(6;11) RCC (Case 2), and (**D**) t(6;11) RCC (Case 3).

Microscopically, Xp11 tRCC is typically comprised of epithelioid cells with clear to eosinophilic cytoplasm that show papillary and nested growth. The tumor cells are large with prominent nucleoli. Psammoma bodies are often observed (Figure 1A). These typical histological characteristics are often observed in Xp11 tRCC with an *ASPSCR1-TFE3* fusion. Xp11 tRCC with a *PRCC-TFE3* fusion shows a smaller structure of nested or papillary cells with less abundant cytoplasm and less conspicuous nuclei when compared with Xp11 tRCC with an *ASPSCR1-TFE3* fusion. Notably, Xp11 tRCCs occasionally share morphological features with clear cell RCC and papillary RCC. Therefore, a definitive diagnosis for Xp11 tRCC cannot be made using morphology alone.

### 4.2. t(6;11) RCC

t(6;11) RCCs do not have a distinctive gross appearance; however, t(6;11) RCCs often show cystic or solid masses and occasionally mahogany-brown cut surfaces.

Microscopically, t(6;11) RCCs typically show a biphasic component, composed of nests of larger epithelioid cells and smaller cells clustered around the basement membrane material. The larger cells have clear to eosinophilic cytoplasm, and the smaller cells have nuclei with condensed chromatin. This characteristic morphology was long considered specific to t(6;11) RCC; however, it is now known that this morphology is occasionally observed in Xp11 tRCCs. Indeed, t(6;11) RCCs are often morphologically diverse without showing the above-mentioned typical morphology [31], suggesting that a thorough analysis is required to correctly diagnose t(6;11) RCC.

Among the three t(6;11) RCC cases that we previously reported [31], one case (Case 1, 57-year-old Japanese man) showed relatively typical morphology (Figure 1B), whereas the other two cases resembled clear cell RCC (Case 2, 37-year-old Japanese man, Figure 1C) and chromophobe RCC (Case 3, 47-year-old Japanese man, Figure 1D). In the latter two cases, there were no morphological characteristics of MiT family tRCC. An expanded spectrum of t(6;11) RCC has also been reported by others [32,33]. Therefore, when encountering an RCC that has an atypical morphology not usually observed in common RCCs, MiT family tRCC must be included in the differential diagnosis. Recent reports introduced sclerosing *TFEB*-rearranged RCC [33,37]. One example case reported by Argani’s group demonstrated an extensively sclerotic and ossified *TFEB*-rearranged RCC (37-year-old man) [33]. Another case reported by Williamson et al. demonstrated a *TFEB*-rearranged RCC (54-year-old man) composed of fibrosis, hyalinization, calcification, ossification, and a smaller component of epithelioid cells. This case was immunohistochemically positive for cytokeratin AE1/AE3. Therefore, pathologists should be aware that an extensively sclerotic morphological pattern potentially represents a recurring histology of *TFEB*-rearranged RCC.

## 5. Immunohistochemical Characteristics of MiT Family tRCC

**Figure 2:**
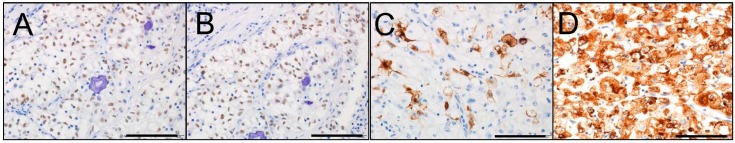
Immunohistochemistry of MiT family tRCC. (**A**) TFE3 staining for Xp11 tRCC, (**B**) TFEB staining for t(6;11) RCC, (**C**) HMB45 staining for t(6;11) RCC, and (**D**) Cathepsin K staining for t(6;11) RCC. Scale bar, 100 µm.

TFE3 and TFEB are overexpressed in the nuclei of Xp11 tRCCs and t(6;11) RCCs, respectively. Thus, Xp11 tRCC and t(6;11) RCC can be immunostained for TFE3 and TFEB, respectively [31,38]. The specificity of nuclear immunostaining for TFE3 in Xp11 tRCC (Figure 2A) and TFEB in t(6;11) RCC (Figure 2B) is high. However, tissues for TFE3 and TFEB immunostainings are susceptible to fixation and other processing methods that can yield false-positive and false-negative results. My experience suggests that diffuse positive immunostainings for TFE3 and TFEB in the tumor are rare, with most cases exhibiting a heterogeneous immunostaining even within in the same case. In our hands, even an anti-TFE3 monoclonal antibody sometimes resulted in the false-positive immunostaining of normal renal tubules and glomeruli. These false-positive or false-negative results may be the result of fixation and immunostaining conditions and the formalin-fixed paraffin-embedding (FFPE) process.

The most specific antibodies for Xp11 tRCC and t(6;11) RCC are the anti-TFE3 antibody and anti-TFEB antibody, respectively. However, an immunohistochemical panel (Table 1) that includes multiple antibodies can be useful to diagnose MiT family tRCC. Because the MiT family are transcription factors that play a role in melanocyte or osteoclast differentiation, MiT family tRCCs often express melanosome-related antigens that are positive for HMB45 (Figure 2C) and/or Melan A and Cathepsin K (Figure 2D), which is expressed in osteoclasts. Therefore, positive immunostainings for HMB45, Melan A, and Cathepsin K are often useful to identify MiT family tRCC [32,39,40]. For Xp11 tRCCs, the immunoreactivity of Cathepsin K differs according to the fusion partner of *TFE3* [41,42]. Xp11 tRCC with a *PRCC-TFE3* fusion shows a higher rate of Cathepsin K positivity (86%, 12 out of 14 cases), whereas all of the cases of Xp11 tRCC with an *ASPSCR1-TFE3* fusion were negative for Cathepsin K (0%, 0 out of 8 cases) [41]. On the other hand, all the cases of t(6;11) RCCs (100%, 7 out of 7 cases) showed strong and diffuse cytoplasmic immunostaining for Cathepsin K [43].

Evidence shows that the immunohistochemical characteristics of MiT family tRCCs are diverse. MiT family tRCCs are typically negative for EMA, cytokeratin AE1/AE3, and CK7; however, we identified a case of t(6;11) RCC (Case 3) with positive staining for these three markers [31]. Argani’s group also reported the cytokeratin labeling in t(6;11) RCCs [32].

## 6. How to Diagnose MiT Family tRCC

The frequency of MiT family tRCC in clinical practice appears to be much lower than that reported in the literature. One explanation is that many MiT family tRCCs may not exhibit a typical morphology but rather show morphologies uncommon to RCCs, such as clear, papillary, and chromophobe RCCs. A molecular examination of RCCs with uncommon morphologies would likely increase the frequency of MiT family tRCC. Another explanation for this low frequency is the technical difficulty of immunostaining for TFE3 and TFEB. Antibodies with high specificity and sensitivity against TFE3 or TFEB are needed.

There are three approaches to diagnose MiT family tRCC: immunohistochemistry, break-apart fluorescence in situ hybridization (FISH), and reverse transcriptase-polymerase chain reaction (RT-PCR)/5′-rapid amplification of cDNA ends (5′-RACE)/karyotyping. The former two approaches can be applied to FFPE specimens, whereas the latter approach usually requires fresh specimens.

### 6.1. Immunohistochemistry

Immunohistochemically, Xp11 tRCC and t(6;11) RCC are positive for TFE3 and TFEB, respectively. However, results must be cautiously interpreted because of the false-positive or false-negative results caused by the technical issues of fixation and immunostaining. Thus, immunohistochemical analyses for MiT family tRCC should utilize an immunohistochemical panel (Table 1). Recent evidence shows that the immunohistochemical positivity for TFE3 or TFEB in the nuclei does not directly provide a diagnosis of Xp11 tRCC or t(6;11) RCC because these proteins can be overexpressed in nuclei by mechanisms other than genetic fusion. One of these mechanisms is chromosome amplification. Indeed, RCCs with TFE3 or TFEB overexpression caused by genetic or chromosome amplification were reported to behave aggressively with a poor prognosis [26,27,28,44]. Furthermore, RCCs with *ALK* fusion often show positive nuclear TFE3 immunostaining by the method using automated immunostaining machine [45]. For TFE3, the assay done manually using an overnight incubation with the antibody (clone p16, Santa Cruz Biotechnology, Santa Cruz, CA, USA), which is the method used in the initial description of this validated assay [38], is superior to the assay done using semiautomated immunostaining machine [21]. For TFEB, the assay using the polyclonal antibody (catalog no. sc-11004, Santa Cruz Biotechnology) was validated [46]. For Cathepsin K, the assay using the antibody (clone 3F9, Abcam, Cambridge, UK) was validated [41].

### 6.2. Break-Apart FISH

When MiT family tRCC is suspected, break-apart FISH can be used to detect fusions of *TFE3* or *TFEB* in FFPE specimens [33,39,47]. Break-apart FISH for *TFE3* or *TFEB* avoids issues related to PCR amplification and is easier to conduct than RT-PCR. However, false-negative results using common break-apart FISH probes in Xp11 tRCC with an *RBM10-TFE3* or *NONO-TFE3* fusion were reported [48,49]. *RBM10* is located at Xp11.23, only 1.8 Mb from *TFE3*. *NONO* is located at Xq13.1, also near *TFE3*. Therefore, performing FISH with specific probes for *RBM10-TFE3* and *NONO-TFE3* fusions may be necessary [48,49].

### 6.3. RT-PCR, 5′-RACE, and Karyotyping

When a fresh specimen is available, RT-PCR, 5′-RACE, or karyotyping can be used to demonstrate the fusion of *TFE3* or *TFEB*. RT-PCR for Xp11 tRCC assumes the fusion partner of *TFE3*. Common *TFE3* fusion partners are *ASPSCR1*, *PRCC*, and *SFPQ*. If the fusion gene is not detected by RT-PCR, 5′-RACE can be used to identify the fusion partner. Karyotyping (Q-banding or G-banding) can also be used to identify the translocation.

**Figure 3:**
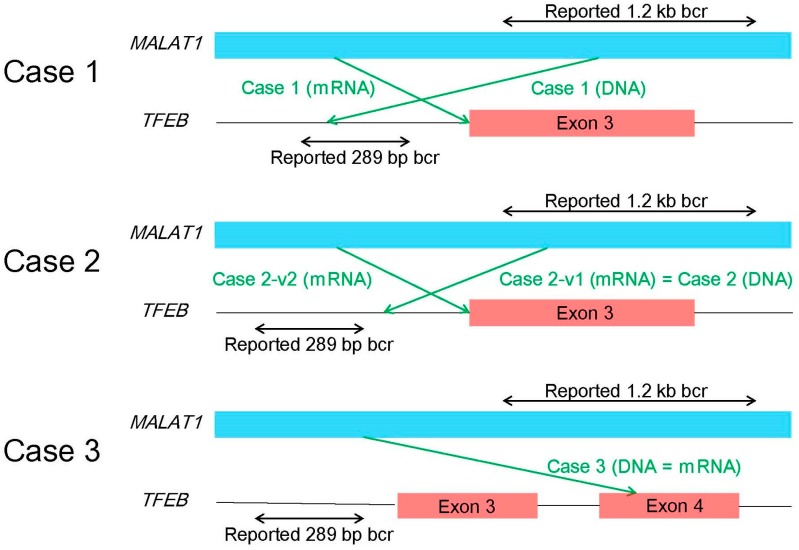
Schematic representation of each fusion pattern for three cases (Cases 1‒3) of t(6;11) renal cell carcinoma. bcr: breakpoint cluster region.

A diagnosis of t(6;11) RCC requires caution. In the original cases reported by Argani’s group [46], the breakpoint of *MALAT1* is localized in a 1.2-kb region, whereas the breakpoint of *TFEB* is localized in a 289-bp region upstream of the translation initiation codon ATG in exon 3. Argani’s group conducted both PCR and RT-PCR for three cases of t(6;11) RCC. All three PCR products matched the corresponding RT-PCR products. Therefore, PCR using DNA was considered to be sufficient to make a diagnosis of t(6;11) RCC. However, our analyses revealed more diverse fusion patterns in t(6;11) RCC as shown in Figure 3 [31]. In Case 1, the breakpoint of DNA was different from that of mRNA, which may be due to modifications such as splicing prior to the formation of mRNA. In Case 2, there were two mRNA products, with the longer one being identical to the DNA product. In Case 3, the breakpoint of *TFEB* existed in exon 4, downstream of exon 3, including the translation initiation codon ATG. However, this case was also immunohistochemically positive for nuclear TFEB, and the size of the TFEB protein was nearly the same as wild-type TFEB by western blotting. As mentioned above, the *MALAT1-TFEB* fusion has been demonstrated to be more complex than originally thought.

## 7. RCC with Chromosome 6p Amplification

Recently, RCC with 6p amplification was identified as an RCC with characteristic histology and aggressive behavior [26,27,28]. Argani et al. reported eight cases of *TFEB*-amplified RCC (six without *TFEB* rearrangement and two with *TFEB* rearrangement) [26]. Although all *TFEB*-amplified RCCs showed aberrant melanocytic marker expression, *TFEB*-amplified RCCs were different from t(6;11) RCC in some ways. For example, *TFEB*-amplified RCC occurred in older patients (median age, 64.5 years) when compared with unamplified t(6;11) RCC (median age, 31 years). Morphologically, *TFEB*-amplified RCC frequently shows nests of high-grade epithelioid cells with pseudopapillary formation and necrosis or true papillary formations. TFEB protein expression was immunohistochemically detectable in six out of eight cases. Importantly, *TFEB*-amplified RCCs were associated with a more aggressive clinical course, whereas t(6;11) RCCs are usually indolent. Although three new partners of *TFEB* were identified in TCGA datasets of RCC, all the three cases were RCC with 6p amplification, and thus likely passenger fusions [25,27]. Gupta et al. identified 25 cases of *TFEB*-amplified RCC [28]. All cases had associated amplifications of *VEGFA* (which exists in 6p21, same with *TFEB*) and occurred in adults (mean age, 66 years). Most of these cases morphologically showed oncocytic and tubulopapillary features with high-grade nuclei, and their clinical courses were aggressive with metastasis and death from RCC in 46% of cases. Although these tumors have *VEGFA* amplification, there is very little evidence at this point that the co-amplification of *VEGFA* in some cases has any therapeutic impact. Additional studies are required to determine whether anti-VEGF therapy is effective in these patients.

## 8. *ALK*-rearranged RCC

*ALK*-rearranged RCC was recently identified. In 2011, RCCs with *VCL-ALK* fusion in young patients with sickle cell traits were reported [50,51]. Because the original RCCs with *VCL-ALK* fusion showed morphological features similar to those in renal medullary carcinoma, they were incorrectly classified as renal medullary carcinoma. RCCs with *VCL-ALK* fusion appear to be different from other *ALK*-rearranged RCCs not associated with *VCL-ALK* fusion; RCCs with *VCL-ALK* fusion are specifically associated with sickle cell traits, typically occur in young patients, and morphologically have more striking vacuolization of the cytoplasm [52]. In 2012, among unclassified RCCs, an RCC with *TPM3-ALK* fusion (36-year-old Japanese woman) and RCC with *EML4-ALK* fusion (53-year-old Japanese woman) were identified [53]. Morphologically, these two cases demonstrated a papillary component, mucinous cribriform pattern, and solid component with rhabdoid cells. In 2016, two cases of RCC with an *STRN-ALK* fusion (33-year-old and 38-year-old Japanese women) were reported [54]. Morphologically, these two cases demonstrated solid, papillary, tubular, and mucinous cribriform structures with psammoma bodies. The tumor cells had large nuclei with prominent nucleoli and eosinophilic cytoplasm, including rhabdoid or signet-ring cell features. The morphology of these RCCs with an *STRN-ALK* fusion was similar to that of the *ALK*-rearranged RCCs reported in 2012 [53], and different from RCCs with a *VCL-ALK* fusion [50,51]. Because *ALK*-rearranged RCCs often show positive nuclear TFE3 immunostaining, the immunohistochemical positivity for TFE3 should be cautiously interpreted [45]. However, the positive TFE3 nuclear labeling identified in these *ALK*-rearranged cases has all been done using automated immunostaining machine [45], which is not the condition with overnight incubation originally described for the identification of TFE3 immunoreactivity [38]. In a survey of more than 500 renal tumors, the overall frequency of *ALK* rearrangement was less than 1% [55]. However, because *ALK*-rearranged RCCs are potentially responsive to ALK inhibitors [56,57,58,59,60,61], every attempt should be made to identify *ALK*-rearranged RCCs.

## 9. Conclusions

Importantly, this review highlights that MiT family tRCCs are clinicopathologically and molecularly diverse. An overview of the recently identified *ALK*-rearranged RCC was also introduced. Notably, newer classifications of RCCs include their molecular features. Indeed, when molecular alterations can be identified, there is an increased chance of treating RCC patients with molecular-targeted therapies. Continued studies on RCC subtypes are needed to better inform the diagnosis and treatment of these cancers.

## Figures and Tables

**Table 1:** Immunohistochemical panel for MiT family tRCC and common RCC subtypes.

RCC subtypes	TFE3	TFEB	Cathepsin K	HMB45	Melan A	CAIX	CK7	AMACR
Xp11 tRCC	+	‒	+/‒	‒/f+	f+/‒	‒/f+	‒	+
t(6;11) RCC	‒	+	+	+/‒	+	‒/f+	‒	+
Clear cell RCC	‒	‒	‒	‒	‒	+	‒	‒
Papillary RCC	‒	‒	‒	‒	‒	‒	+	+/‒
Chromophobe RCC	‒	‒	‒	‒	‒	‒	+	‒

+, positive; f+, focally positive; ‒, negative; RCC, renal cell carcinoma; tRCC, translocation RCC.

## Abbreviations

5′-RACE	5′-rapid amplification of cDNA ends
bHLH	basic helix-loop-helix
FFPE	formalin-fixed paraffin-embedded
FISH	fluorescence in situ hybridization
lncRNA	long non-coding RNA
PEComa	perivascular epithelioid cell tumor
RCC	renal cell carcinoma
RT-PCR	reverse transcriptase-polymerase chain reaction
TCGA	The Cancer Genome Atlas
tRCC	translocation renal cell carcinoma
